# Extraction of Keratin from Pig Nails and Electrospinning of Keratin/Nylon6 Nanofibers for Copper (II) Adsorption

**DOI:** 10.3390/polym15020467

**Published:** 2023-01-16

**Authors:** Lanlan Wei, Di Wang, Zhiheng Liao, Zexuan Gong, Wenwen Zhao, Jinyan Gu, Yan Li, Jingjun Li

**Affiliations:** College of Food Engineering, Anhui Science and Technology University, Chuzhou 233100, China

**Keywords:** pig nails, keratin, electrospinning, nanofiber, copper (II) ion adsorption

## Abstract

In this study, keratins were extracted from pig nail waste via the reduction method for the first time, using L-cysteine as the reductant and urea as the lytic agent. Nylon6 and pig nail keratin were successfully combined via electrospinning to generate a series of nylon6/pig nail keratin nanofibers with a variety of keratin concentrations (0% to 8%, *w*/*w*). From the results, it was found that the best concentration was 6% (*w*/*w*). The morphologies of the electrospun nanofibers were examined via scanning electron microscopy (SEM) and transmission electron microscopy (TEM). The structural properties were characterized using Fourier transform infrared spectroscopy (FTIR) and X-ray diffraction (XRD), and the thermal properties were described using thermo-gravimetric analysis (TGA). These results confirmed that the nanofibers were composed of both polymeric phases. Finally, copper (II) was used as a model ion, and the nanofiber membranes exhibited a strong adsorption affinity for metal ions in the water samples. This study provides an important foundation for the application of nanofiber membranes in metal adsorption.

## 1. Introduction

Polymeric nanofibers (NFs) are synthetized via electrospinning, a widely used fabrication technique in which the material is precipitated on a collector [1,2]. As a result of their unique structural properties and functional features, nanofibers have numerous and diverse applications, including wound dressing, drug delivery, vascular tissue engineering, and heavy metal ion adsorption [3,4,5,6,7]. Hence, electrospun nanofibers composed of natural and synthetic polymers have attracted increasing attention in recent years. Although electrospinning is treated as a simple and efficient technique for the production of polymer nanofibers, it cannot be mass-produced to meet industrial needs; bubble-spinning has been proposed and the research has evolved [8]. Keratin is abundant in nature and is a biocompatible and biodegradable material, thus having significant prospects in natural polymer applications [9,10,11]. Keratin is a structural fibrous protein, and is the main component of wool, feathers, nails, horns, and other epithelial coverings [12]. Numerous studies have reported improving the performance of keratin films by mixing keratin with both natural [13] and synthetic polymers [14]. In particular, feather keratin (FK) has been widely used, and FK/PVA composite nanofibers [15] and FK/PVA/PEO three-component nanofiber membranes [16] have been fabricated through an electrospinning process. Pig nail keratin represents an unexplored and potentially useful keratin source, as tons of pig nail waste are discarded every year. Pig nails are rich in proteins and amino acids, and waste represents an important resource. Therefore, the reasonable development of the recycling value of waste pig nails can not only expand its application field, but also meet the development trend of green environmental protection. 

Nylon is a ubiquitous synthetic polymer, and its chemical structure is composed of amide groups separated by methylene sequences. In particular, nylon6 has received significant attention. In its structure, the amide groups are planar because the C-N bond has partial double-bond properties, and the hydrogen bonding of amino and carbonyl groups is maximized in this way [17]. In recent years, researchers have studied the parameters that affect the electrospinning of nylon6 nanofibers [18,19,20]. Moreover, the use of electrospun nylon6 nanofiber membranes as sorbents for heavy metal adsorption has emerged as a popular research topic. Electrospinning is a standard method of fabricating microfibers and nanofibers [21]. These fibers have desirable features, including high porosity, large specific surface area, well-distributed pore size, and outstanding gas exchange characteristics [22].

Copper ion is a heavy metal element and a transition element. It shows Cu^2+^ valence in common compounds [23]. Given their same coordination modes and the stability of transition elements, they are combined with keratin via chelation; the chelation principle is similar to them, and the effect of chelation on high valence metal ions is better [24]. The copper ion solution showed light blue, and in the experiment, the phenomenon was obvious. The content of copper ions in electrochemical wastewater, which pollutes groundwater resources, is very high. The accumulation of heavy metals is harmful to human health. Heavy metals easily accumulate in the brain, kidney, and other organs, leading to progressive damage to the body’s functions [25]. When copper ions enter the human body, they accumulate. When the concentration of copper ions exceeds 2.2 mg, vomiting, poisoning, and liver and kidney damage occur. Based on this background, copper ions are representative of transition metals; copper ion pollution is serious in industry [26]. Numerous techniques, including chemical precipitation, membrane filtration, biological treatment, adsorptions, and photocatalytic reduction, have been developed in the past for Cr(VI) removal [27,28,29,30]. Therefore, copper ions were selected as a heavy metal representative; the capacity to adsorb copper ions was used as the evaluation index to compare the adsorption capacity of different types of membranes and to evaluate their application potential as adsorption materials.

In this study, a new bio-based adsorption material for heavy metal ions was developed. Keratin was extracted from pig nail waste; keratin materials are hydrophilic, adsorptive, and able to chelate with metal ions. Thus, nylon6/pig nail keratin composite nanofiber membranes were prepared via electrospinning to adsorb copper ions for the first time. Advantageously, the membranes reused a wasted resource and provide a theoretical basis for the development of keratin-based adsorption materials.

## 2. Materials and Methods

### 2.1. Reagents and Chemicals

Analytical grade chemicals and reagents were used in this study. Urea (CH_4_N_2_O), L-cysteine (C_3_H_7_NO_2_S, molecular mass: 121.16), sodium hydroxide (NaOH), hexafluoroisopropanol (C_3_H_2_F_6_O), gelatin (gel, gel strength ~250 g, Bloom), and glycerol (gly, molecular mass: 92.09) were purchased from Shanghai Macklin Biochemical Company (Shanghai, China, 15 May 2021). Hydrochloric acid (HCl) and nylon6 were purchased from DongQi Biotechnology Co., Ltd. (Shenzhen, China).

### 2.2. Electrospinning of Nylon6/Pig Nail Keratin Nanofibers

**Figure 1:**
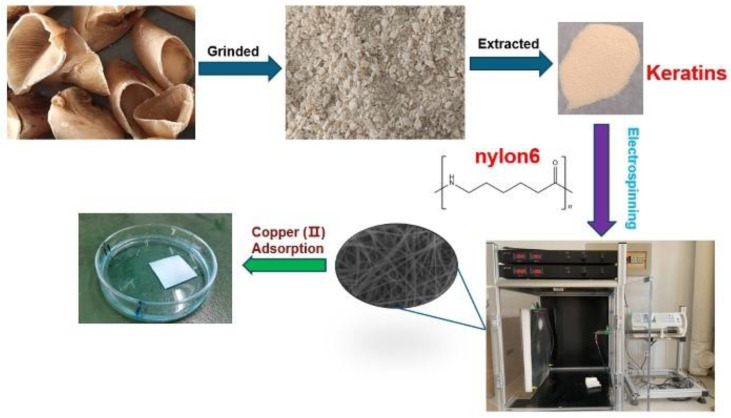
Schematic of the extraction of keratin from pig nails, the preparation of keratin/nylon6 nanofibers via electrospinning, and the application of the nanofiber membrane for the adsorption of copper (II).

As shown in Figure 1, 3.0 g of pig nails was extracted in 4 mol L^−1^ urea, and 0.45 g L-cysteine was added. The aqueous solution was shaken well at 150 rpm for 16 h at 70 °C. Subsequently, the extract was centrifuged at 10,000 rpm for 15 min, and the pH of the supernatant was adjusted to 4.0 with 0.2 mol L^−1^ HCl to precipitate the keratin. The precipitate was washed using deionized water and freeze dried in a vacuum freeze dryer to obtain pig nail keratin powder.

A 10% nylon6 solution was prepared by dissolving nylon6 in hexafluoroisopropanol (HFIP). To the nylon6 solution, 2 wt.%, 4 wt.%, 6 wt.%, and 8 wt.% of pig nail keratin were added to prepare four different keratin solutions. The four mixed solutions were sonicated for approximately 1.0 h to facilitate the homogeneous dispersion of nylon6 and pig nail keratin. A rectangular foam board covered with aluminum foil was used to collect the nanofibers. The blended solutions were each loaded into a 10 mL glass syringe with a blunt 23–gauge needle attached. A syringe pump, at a flow rate of 1.0 mL min^−1^ and a constant positive voltage of 25 kV, was applied to each needle. The distance between the needle tips and the collecting plates was 12–15 cm. The electrospinning experiments were carried out at room temperature.

### 2.3. Scanning Electron Microscopy

The surface morphologies of the electrospun fibers were observed using a scanning electron microscope (SEM, EVO–18, ZEISS, Oberkochen, Germany) at 20 kV. The average diameter of the fibers was determined by analyzing 30 individual fibers using Nano measurer software 1.2 (China).

### 2.4. Transmission Electron Microscopy

The nanofiber samples were deposited onto copper grids and photographed using a transmission electron microscope (TEM, JEM–100CX, JEOL, Tokyo, Japan). 

### 2.5. Thermogravimetric Analysis

The nanofibers’ thermal stability was analyzed via a thermogravimetric analyzer (TGA, TAQ600, NETZSCH–Gerätebau GmbH, Selb, Germany) under a nitrogen atmosphere. The temperature of the experiment ranged from 25–800 °C at a rate of 10 °C min^−1^.

### 2.6. Fourier Transform Infrared Spectroscopy

The structure of the nanofiber membranes was confirmed using FTIR (FTIR–850, Tianjin Gangdong, Tianjin, China), using a range of 4000–500 cm^−1^.

### 2.7. X-ray Diffraction Analysis

X-ray diffraction analysis was carried out on an X-ray diffractometer (XRD–3, Beijing Purkinje General, Beijing, China), and the XRD spectra were recorded in the 2θ range of 5° to 60° at a step rate of 0.02°.

### 2.8. Tensile Properties

The tensile properties of the nanofibers, including the tensile strength and elongation at break, were measured using a universal testing machine (CMT6503, Shenzhen MTS Test Machine Company Ltd., Shenzhen, China), using the ASTM standard D638 with a rate of 5.0 mm min^−1^. Films were cut to 40 mm × 5.0 mm. The thicknesses of the samples were measured via a micrometer, and ranged from 0.09 to 0.12 mm for the various conditions studied. Three replications of each formulation were performed, and the average values were calculated.

### 2.9. The Experiment of Adsorption Performance

Fifty milliliters of copper ion standard solution (1.0 mg L^−1^) was transferred to each of five 100 mL conical flasks, and the pHs were adjusted to 4.0 using HNO_3_ solution. Next, 30 mg keratin/nylon6 nanofiber membrane was added to each conical flask, and the flasks were placed in a constant temperature water bath at room temperature and shaken at 150 rpm for 1.0 h. Following this, the contents of five metal ions were measured using an atomic absorption spectrometer (ABS); the adsorptions were calculated via the following formula [31]:

$$q_{e}=\frac{\left(c_{0}-c_{e}\right)V}{M}$$

where $c_{0}$ is the concentration of five ions in the mixed solution before adsorption (mg L^−1^); $c_{e}$ is the concentration of five ions in the mixed solution after adsorption (mg L^−1^); *V* is the volume of the mixed solution (L); and *M* is the mass of the keratin/nylon6 nanofiber membrane (mg).

## 3. Results

### 3.1. Morphological Characterization of Pig Nail Powders and Pig Nail Keratin

**Figure 2:**
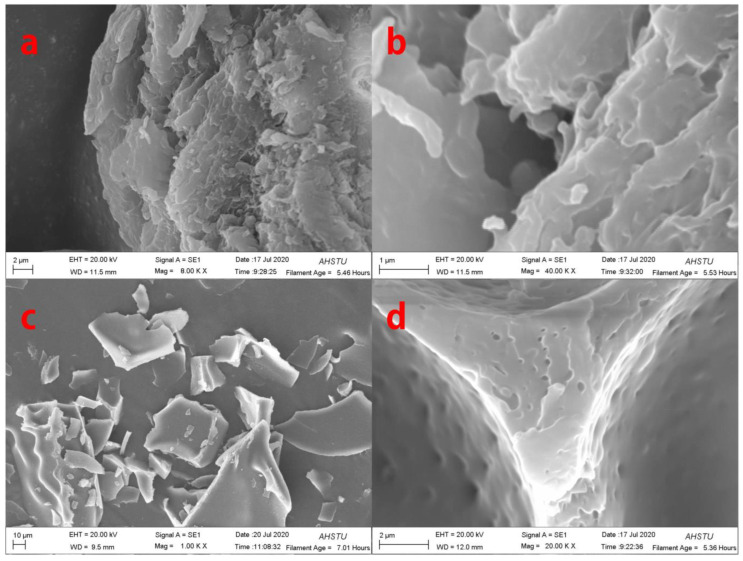
SEM images of (**a**,**c**) pig nail powders; and (**b**,**d**) pig nail keratin.

To understand the morphological changes in pig nail powders and pig nail keratin intuitively, they were analyzed using SEM. The external structure of the undissolved pig nail powder was arranged neatly, as shown in Figure 2a,c. However, upon dissolution, intermolecular forces and chemical bonds were destroyed, leading to changes in the morphological structure of pig nail powder in which the keratin was shed from the pig nails (Figure 2b,d).

### 3.2. Morphological Characterization of Electrospun Nylon6/Pig Nail Keratin Nanofibers

**Figure 3:**
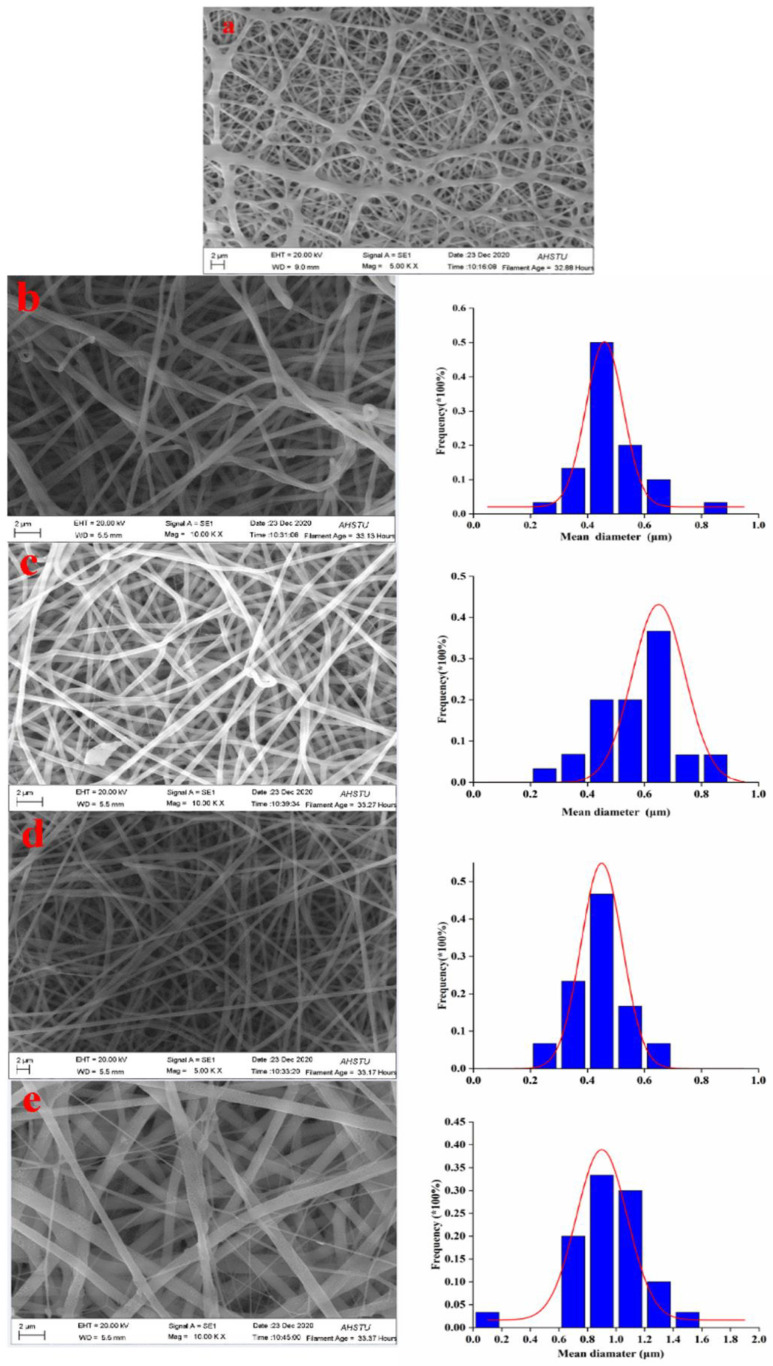
SEM images and diameter distributions of: (**a**) 12% nylon6 nanofiber membrane; (**b**) 12% nylon6/2% pig nail keratin composite; (**c**) 12% nylon6/4% pig nail keratin composite; (**d**) 12% nylon6/6% pig nail keratin composite; and (**e**) 12% nylon6/8% pig nail keratin composite.

**Figure 4:**
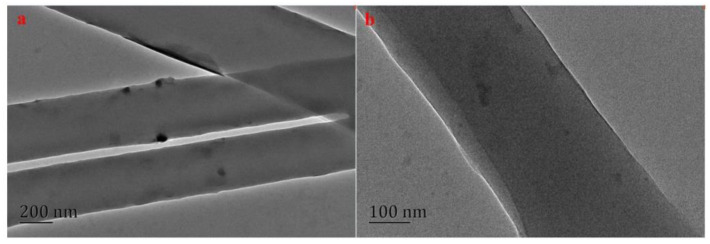
TEM images of 12% nylon6/6% pig nail keratin composite, scale bar: (**a**) 200 nm; (**b**) 100 nm.

Figure 3 shows SEM images and the corresponding nanofiber diameter distributions of the electrospun composite nanofibers with various concentrations of pig nail keratin (ranging from 0 to 8.0 wt.%). As shown in Figure 4, almost all the electrospun nylon6 nanofibers were stuck together, and no obvious structure was observed. Hence, the diameters of nylon6 nanofibers were not measured; the nanofiber diameters changed with the addition of pig nail keratin. When 2.0 wt.% pig nail keratin was added to the nylon6 solution, the 12% nylon6/2.0% keratin nanofibers became more curved. In addition, the nanofiber diameters were thicker and varied (220–870 nm), with an average diameter of approximately 470 nm. The diameters of the 12% nylon6/4.0% keratin nanofibers also varied (290–820 nm, average 580 nm), and the majority of 12% nylon6/6.0% keratin nanofibers had diameters of 290–650 nm (average 430 nm). The diameters of 12% nylon6/8.0% keratin nanofibers were coarser, with diameters from 610 to 1430 nm, with an average of approximately 940 nm. Generally, 12% nylon6/6.0% keratin nanofibers were the thinnest. The average diameter of the nylon6/pig nail keratin nanofibers initially decreased as the keratin content increased to 6.0%. At 8.0 wt.%, the diameter increased, and the viscosity of the electrospinning solution increased. Differences in the nanofiber diameters were likely due to differences in the conductivities and viscosities of the electrospinning solutions [32].

Figure 4 shows TEM images of the electrospun 12% nylon6/6% keratin composite nanofibers. The surfaces of the nanofibers were smooth and uniform, and some pig nail keratin particles were exposed on the surfaces of nanofibers. This result indicates that the compatibility of the nylon6 and keratin in the solvent was excellent. Moreover, the smooth nanofiber surface was beneficial to the adsorption of target analytes [33]. 

### 3.3. X-ray Diffraction (XRD) Analysis of Electrospun Nylon6/Pig Nail Keratin Nanofibers

**Figure 5:**
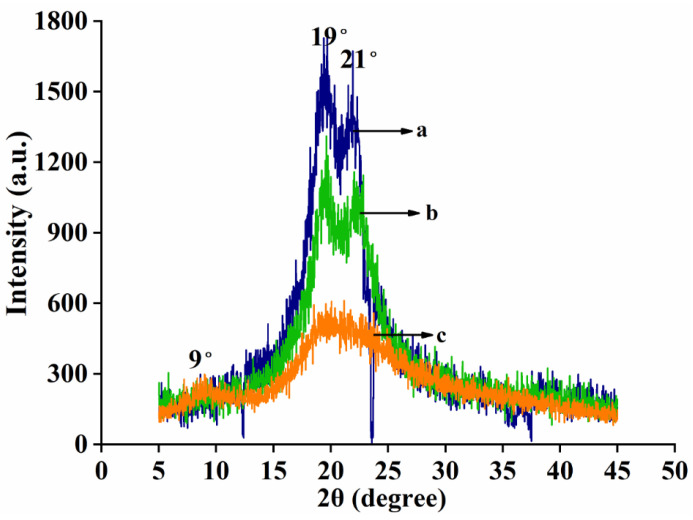
XRD patterns of (a) nylon6 nanofiber membranes; (b) nylon6/pig nail keratin composite nanofiber membranes; and (c) pure pig nail keratin.

X-ray diffractograms of nylon6, pig nail keratin, and electrospun nanofiber membranes are shown in Figure 5. There were obvious diffraction peaks for nylon6 at 2θ = 19° and 22°, but there was no diffraction peak for keratin. The XRD pattern obtained for pure pig nail keratin exhibited broad diffraction peaks at 9° and 20°, which are characteristic of partially crystalline materials. Sharp diffraction peaks at 19° and 21° were observed for nylon6, indicating high crystallinity. For the nylon6/pig nail keratin nanofibers, diffraction peaks were observed at 20° and 22° [34].The intensity of these peaks was lower than that of the peaks observed for nylon6, and there was no diffraction peak at 9°. The results suggest that the overall crystallinity of the nanofiber composites was slightly lower than that of nylon6 due to the addition of keratin, which contains a small crystalline region and a large amorphous region.

### 3.4. Thermal Properties of Nanofibers

**Figure 6:**
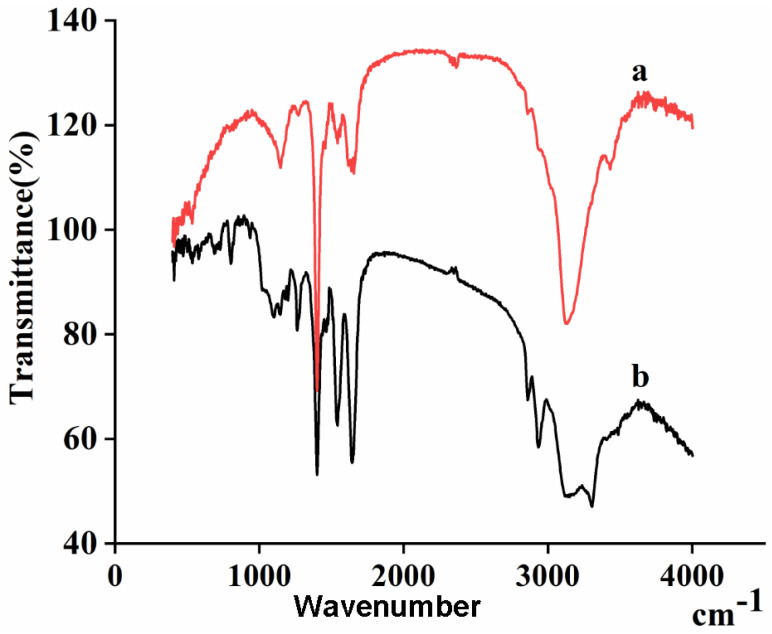
Infrared spectra of (a) nylon6 nanofiber membranes and (b) nylon6/pig nail keratin composite nanofiber membranes.

**Figure 7:**
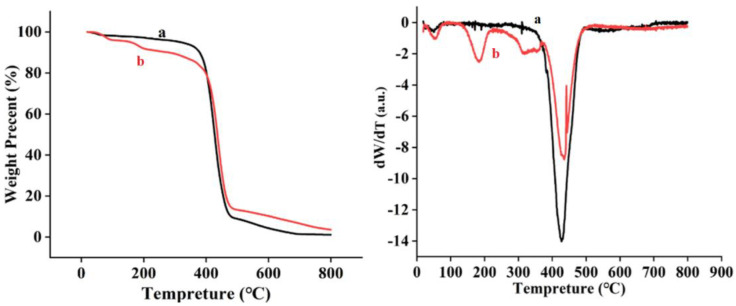
TGA curves and derivative thermogravimetric (DTG) curves for the (a) nylon6 nanofiber membranes and (b) nylon6/pig nail keratin composite nanofiber membranes.

The thermal stability of the electrospun nanofibers was determined via thermal analyses. As shown in Figure 6, the thermogravimetric thermograms (TGA) of the nanofibers were compared with derivative thermogravimetric (DTG) diagrams. Two-step degradation was observed in all samples. The initial weight loss of nylon6 and nylon6/pig nail keratin nanofibers took place at 70–400 °C, due to moisture loss. The second weight loss took place in the temperature range of 400–480 °C, and was due to the thermal degradation of the nylon6 and the nylon6/pig nail keratin matrix. As shown in Figure 7, the weight loss of nylon6 was lower than that of nylon6/pig nail keratin nanofibers between 70–400 °C. However, nylon6/pig nail keratin nanofibers showed higher thermal stabilities than pure nylon6 between 400–480 °C. The results demonstrate that the addition of keratin provided effective reinforcement against the thermal degradation of the polymer matrix, increasing the thermal stability of the matrix. The maximum thermal degradation temperatures were 421 °C and 438 °C. 

### 3.5. Structural Analysis

As shown in the FTIR spectra in Figure 7, a unique adsorption of peptide bonds (–CONH–) was discovered, which was characteristic of pig nail keratin and nylon6 nanofibers. The amide I bands at 1647 cm^−1^ were attributed to the C=O stretching vibration. The amide II bands, due to N–H bending vibrations, were observed at 1548 cm^−1^. Moreover, a sharp peak at 1265 cm^−1^ corresponded to the characteristic absorption peak of the amide III bands (1220–1300 cm^−1^). The adsorption peak at 3304 cm^−1^ was ascribed to the stretching vibrations of N–H bonds. The absorption peaks of the two samples were similar, but the intensity decreased with the addition of pig nail keratin. This result confirms the successful synthesis of nylon6/pig nail keratin nanofiber membranes.

### 3.6. Mechanical Characterization

**Figure 8:**
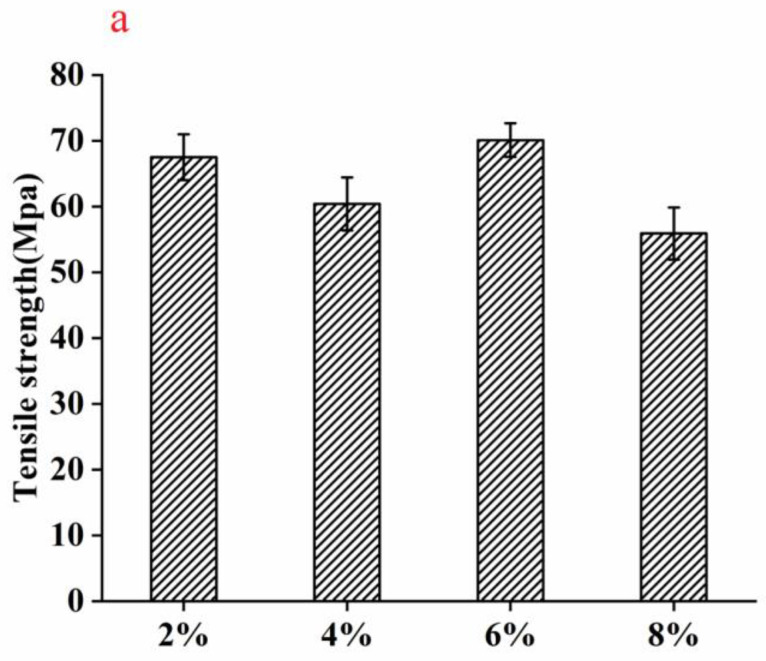

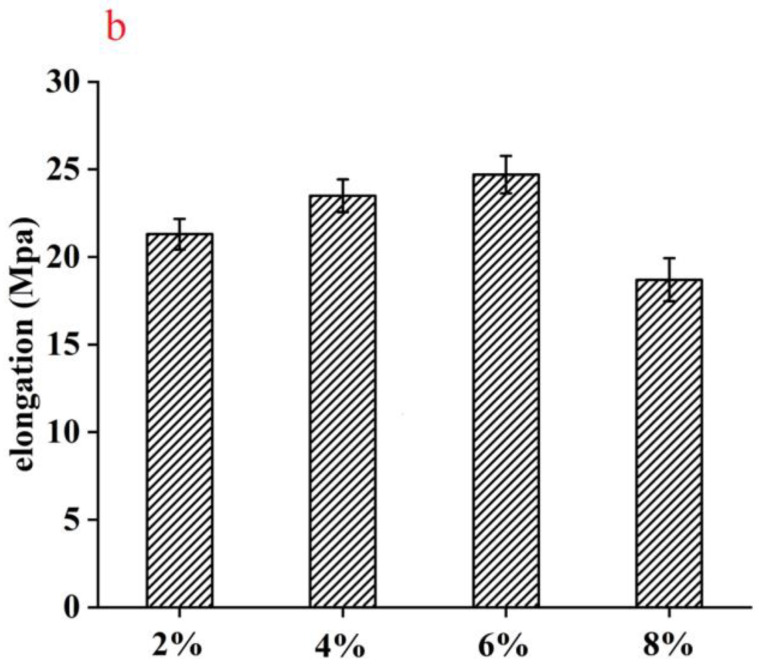
Mechanical properties of nylon6/pig nail keratin nanofiber membranes, including (**a**) tensile strength and (**b**) elongation.

The mechanical properties of the nylon6/pig nail keratin nanofiber membranes are presented in Figure 8. The mechanical properties, including tensile strength and elongation, changed with the addition of pig nail keratin. The tensile strength and elongation of 12% nylon6/6.0% pig nail keratin nanofiber membrane were 70.1 MPa and 24.7 MPa, respectively, which were stronger than those of the 2.0%, 4.0%, and 8.0% keratin membranes. In addition to the excellent compatibility and the interaction between pig nail keratin and nylon6, the strength of the membrane was enhanced due to an increase in β–sheet structures with increasing keratin concentration. As α-helix cannot supply a rigid structure compared with β–sheet structure, the β–sheet structure resulted in the materials own high strength and small displacement performance [35].

### 3.7. Adsorption Performance of Nylon6/Pig Nail Keratin Nanofiber Membranes

**Figure 9:**
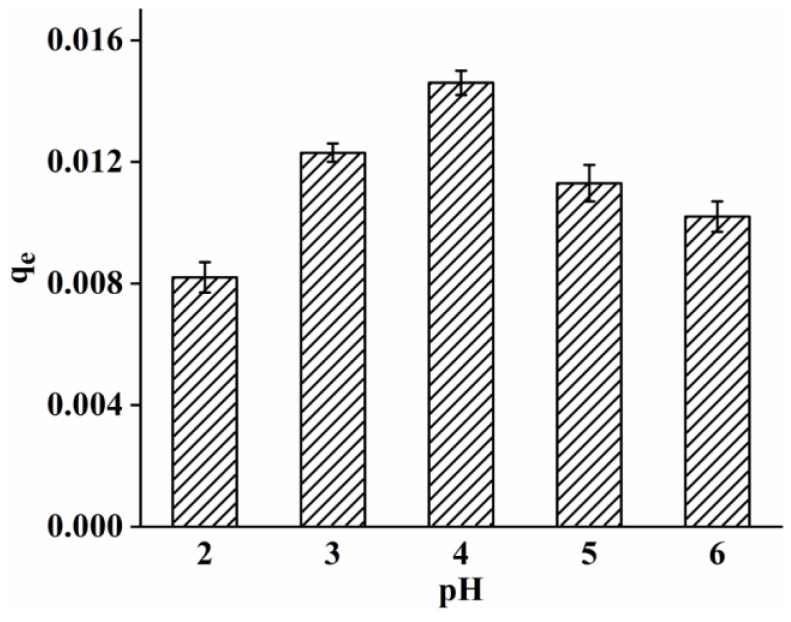

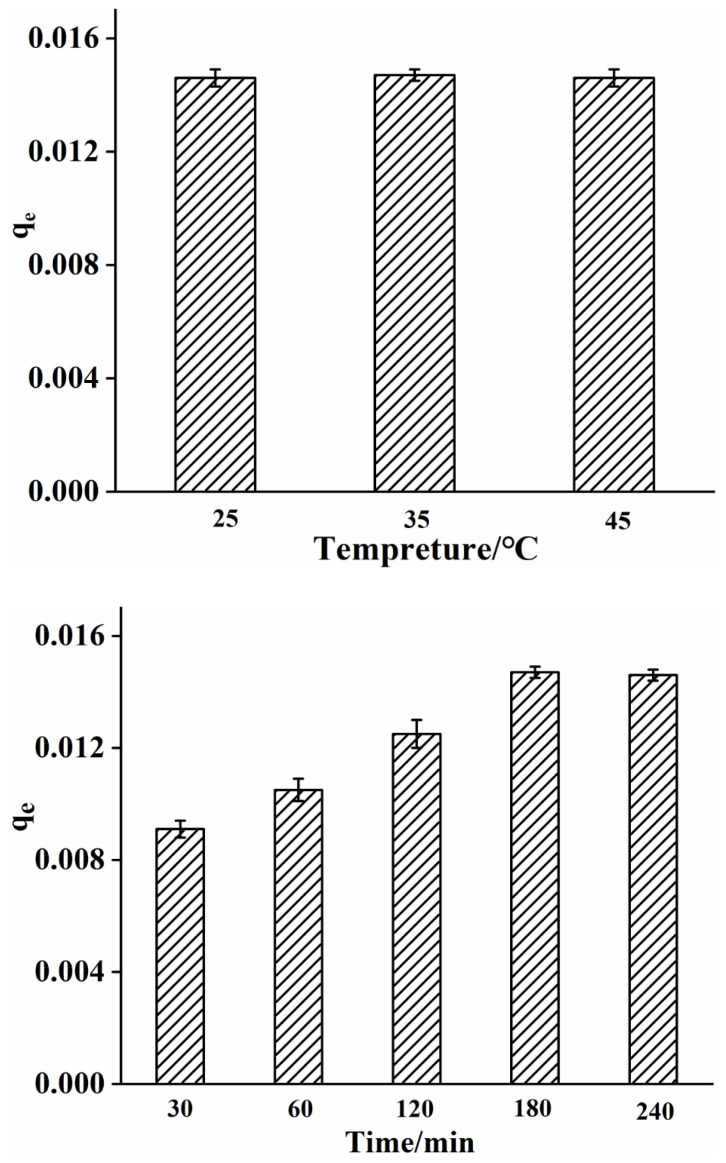
Adsorption performance of nylon6/pig nail keratin nanofiber membranes under different concentrations.

As shown in Figure 9, with increasing pH, from pH 2–4, the adsorption capacity increased and reached its maximum at pH 4. When the pH was low, there was much H^+^ in the result, and H^+^ occupied the limited adsorption unit on the adsorbent, as competitive adsorption exists between heavy metal ions. Under the most suitable pH conditions, the adsorption strength of keratin/nylon6 nanofibers gradually increased as the reaction time increased, and the adsorption equilibrium was achieved in 180 min. With increasing temperature, from 25–45 °C, the metal ions adsorption capacity of keratin/nylon6 nanofiber membrane was not significantly different, indicating that temperature had no effect on the adsorption capacity.

## 4. Conclusions

Herein, we demonstrated the successful synthesis of nylon6/pig nail keratin nanofiber membranes using a novel electrospinning process. Pig nail keratin was extracted and combined with nylon6 via electrospinning. The average diameter of the nanofibers changed from 470 ± 36 nm to 580 ± 58 nm, 430 ± 45 nm, and 980 ± 65 nm for different concentrations of pig nail keratin (ranging from 0 to 8.0 wt.%). Similarly, the thermal stability of the nylon6/pig nail keratin nanofiber membranes significantly improved. The tensile strength and elongation of 12% nylon6/6.0% pig nail keratin nanofiber membranes were 70.1 MPa and 24.7 MPa, respectively, which were higher than those of the other keratin nanofiber membranes. The results exhibited excellent compatibility between the individual components, high thermal stability, and good mechanical properties of nylon6/pig nail keratin nanofiber membranes. Moreover, copper (II) ions in water samples were successfully adsorbed by the nanofiber membranes, with high affinity. This study provided important evidence to support the application of nanofiber membranes in metal adsorption.

## Data Availability

Data are contained within the article.

## References

[1] Wang Y., Hu Q., Zhang Y., Jin W., Chu L. (2022). Extraction of garlic essential oil with electrospun nanofibers and its antioxidant activity. Mater. Express.

[2] Chu L., Wang Y., Zhou Y., Kang X. (2021). A novel biosensor based on Blu-ray disc coating film for determination of total amino acid content in tea leaves. Rsc. Adv..

[3] Chang S.K., Gang E.H., Um I.C., Park Y.H. (2007). Nanofibrous membrane of wool keratose/silk fibroin blend for heavy metal ion adsorption. J. Membr. Sci..

[4] Li J., Yi L., Lin L., Mark A., Ko F., Ling Q. (2009). Preparation and biodegradation of electrospun PLLA/keratin nonwoven fibrous membrane. Polym. Degrad. Stabil..

[5] Wu H., Zhong L., Wang X., Liang Z., Mo N. (2014). Lack of association between XPC Lys939Gln polymorphism and prostate cancer risk: An updated meta-analysis based on 3039 cases and 3253 controls. Int. J. Clin. Exp. Med..

[6] Maame B., Nava R., Udhab A., Narayan B. (2015). Fabrication and Characterization of Electrospun PCL-MgO-Keratin-Based Composite Nanofibers for Biomedical Applications. Materials.

[7] Yu D.G., Williams G.R., Wang X., Liu X.K., Li H.L., Bligh S.A. (2013). Dual drug release nanocomposites prepared using a combination of electrospraying and electrospinning. RSC Adv..

[8] Li Y., He J. (2019). Fabrication and characterization of ZrO_2_ nanofibers by critical bubble electrospinning for high-temperature-resistant adsorption and separation. Adsorpt. Sci. Technol..

[9] Zhou Y.F., Zhen G.C., Xiao-Peng L.I., Wei Q.I. (2007). Mix Ratio Design of Membrane Bag Concrete. J. Hebei Eng. Tech. College.

[10] Wu Y., Han C., Yang J., Jia S., Wang S. (2011). Polypropylene films modified by air plasma and feather keratin graft. Surf. Coat. Tech..

[11] Reichl S. (2009). Films based on human hair keratin as substrates for cell culture and tissue engineering. Biomaterials.

[12] Dou Y., Zhang B.N., He M., Yin G.Q., Cui Y.D., Savina I.N. (2015). Keratin/Polyvinyl Alcohol Blend Films Cross-Linked by Dialdehyde Starch and Their Potential Application for Drug Release. Polymers.

[13] Tian Y.K., Dong-Xia L., Xu-Hong Y. (2013). Preparation and properties of keratin/cmc blend membranes. Adv. Mater. Res..

[14] Bertini F., Canetti M., Patrucco A., Zoccola M. (2013). Wool keratin-polypropylene composites: Properties and thermal degradation. Polym. Degrad. Stab..

[15] Ming H., Zhang B., Yao D., Yin G., Cui Y., Chen X. (2017). Fabrication and characterization of electrospun feather keratin/poly (vinyl alcohol) composite nanofibers. RSC Adv..

[16] Ding J., Man C., Chen W., Ming H., Yin G. (2018). Vapor-assisted crosslinking of a FK/PVA/PEO nanofiber membrane. Polymers.

[17] Bortolato S.A., Arancibia J.A., Escandar G.M. (2008). A novel application of nylon membranes to the luminescent determination of benzo[a]pyrene at ultra trace levels in water samples. Anal. Chim. Acta.

[18] Shu Z., Shim W.S., Kim J. (2009). Design of ultra-fine nonwovens via electrospinning of Nylon 6: Spinning parameters and filtration efficiency. Mater. Design.

[19] Ojha S.S., Afshari M., Kotek R., Gorga R.E. (2010). Morphology of electrospun nylon-6 nanofibers as a function of molecular weight and processing parameters. J. Appl. Polym. Sci..

[20] Bazbouz M.B., Stylios G.K. (2010). Alignment and optimization of nylon 6 nanofibers by electrospinning. J. Appl. Polym. Sci..

[21] Mori S. (1976). Identification and determination of phthalate esters in river water by high-performance liquid chromatography. J. Chromatogr. A.

[22] Cai Y.Q., Cai Y., Shi Y.L., Liu J.M., Mou S.F., Lu Y.Q. (2007). A liquid-liquid extraction technique for phthalate esters with water-soluble organic solvents by adding inorganic salts. Microchim. Acta.

[23] Medici V. (2020). Effects of Dietary glucose and fructose on copper, iron, and zinc metabolism parameters in humans. Nutrients.

[24] Chen J., Jiang Y., Shi H., Peng Y., Li C. (2020). The molecular mechanisms of copper metabolism and its roles in human diseases. Pflug. Arch. Eur. J. Phy..

[25] Li X., Xu L., He J. (2020). Nanofibers membrane for detecting heavy metal ions. Therm. Sci..

[26] Collins J.F. (2021). Copper nutrition and biochemistry and human (patho)physiology. Adv. Food Nutr. Res..

[27] Shang G.Y., Zheng R., Ge Q., Feng X., Wang R., Zhou Y., Luo S., Duan L., Lin J., Chen H. (2022). Interfacial engineering of CuFeS_2_ quantum dots via platinum decoration with enhanced Cr (VI) reduction dynamics under UV-Vis-NIR radiation. J. Hazard. Mater..

[28] Chen Z., Wei W., Zou W., Li J., Zheng R., Wei W., Ni B., Chen H. (2022). Integrating electrodeposition with electrolysis for closed-loop resource utilization of battery industrial wastewater. Green Chem..

[29] Zhang Q., Gu H., Wang X., Li L., Zhang J., Zhang H., Li Y., Dai W. (2021). Robust hollow tubular ZnIn_2_S_4_ modified with embedded metal-organic-framework-layers: Extraordinarily high photocatalytic hydrogen evolution activity under simulated and real sunlight irradiation. Appl. Catal. B Environ..

[30] Yu B., Luo J., Xie H., Yang H., Chen S., Liu J., Zhang R., Li Y. (2021). Species, fractions, and characterization of phosphorus in sewage sludge: A critical review from the perspective of recovery. Sci. Total Environ..

[31] He M., Zhang B., Dou Y., Yin G., Cui Y. (2016). Blend modification of feather keratin-based films using sodium alginate. J. Appl. Polym. Sci..

[32] Saeed K., Haider S., Oh T.J., Park S.Y. (2008). Preparation of amidoxime-modified polyacrylonitrile (pan-oxime) nanofibers and their applications to metal ions adsorption. J. Membrane Sci..

[33] Tian D., Li X., He J. (2019). Geometrical potential and nanofiber membrane’s highly selective adsorption property. Adsorpt. Sci. Technol..

[34] Zhang H., Wang J., Yu N., Liu J. (2014). Electrospun PLGA/multi-walled carbon nanotubes/wool keratin composite membranes: Morphological, mechanical, and thermal properties, and their bioactivities in vitro. J. Polym. Res..

[35] Ayutthaya S.I.N., Tanpichai S., Wootthikanokkhan J. (2015). Keratin extracted from chicken feather waste: Extraction, preparation, and structural characterization of the keratin and keratin/biopolymer films and electrospuns. J. Polym. Environ..

